# Transcranial Magnetic Stimulation of the Supplementary Motor Area in the Treatment of Obsessive-Compulsive Disorder: A Multi-Site Study

**DOI:** 10.3390/ijms17030420

**Published:** 2016-03-22

**Authors:** Emily R. Hawken, Dancho Dilkov, Emil Kaludiev, Selcuk Simek, Felicia Zhang, Roumen Milev

**Affiliations:** 1 Departments of Psychiatry, Queen’s University, Kingston, ON K7L 4X3, Canada; erhawken@gmail.com (E.R.H.); f.i.zhang67@gmail.com (F.Z.); 2 Biomedical and Molecular Sciences, Queen’s University, Kingston, ON K7L 4X3, Canada; 3 Department of Psychiatry, Military Medical Academy, Sofia 1000, Bulgaria; ddilkov@abv.bg (D.D.); ekaludiev@abv.bg (E.K.); 4 Department of Psychiatry, Sincan State Hospital, Istanbul 34400, Turkey; selcuk_simsek@yahoo.com

**Keywords:** Yale-Brown obsessive compulsive scale, sensory motor area, low-frequency rTMS

## Abstract

Recently, strategies beyond pharmacological and psychological treatments have been developed for the management of obsessive-compulsive disorder (OCD). Specifically, repetitive transcranial magnetic stimulation (rTMS) has been employed as an adjunctive treatment in cases of treatment-refractory OCD. Here, we investigate six weeks of low frequency rTMS, applied bilaterally and simultaneously over the sensory motor area, in OCD patients in a randomized, double-blind placebo-controlled clinical trial. Twenty-two participants were randomly enrolled into the treatment (ACTIVE = 10) or placebo (SHAM = 12) groups. At each of seven visits (baseline; day 1 and weeks 2, 4, and 6 of treatment; and two and six weeks after treatment) the Yale-Brown Obsessive Compulsive Scale (Y-BOCS) was administered. At the end of the six weeks of rTMS, patients in the ACTIVE group showed a clinically significant decrease in Y-BOCS scores compared to both the baseline and the SHAM group. This effect was maintained six weeks following the end of rTMS treatment. Therefore, in this sample, rTMS appeared to significantly improve the OCD symptoms of the treated patients beyond the treatment window. More studies need to be conducted to determine the generalizability of these findings and to define the duration of rTMS’ clinical effect on the Y-BOCS. Clinical Trial Registration Number (NCT) at www.clinicaltrials.gov: NCT00616486.

## 1. Introduction

Obsessive compulsive disorder (OCD) is a chronic and debilitating psychiatric illness that has been reported to affect 1%–3% of the world population [1,2]. Considered a heterogeneous condition, its clinical features consist mainly of obsessions (*i.e*., persistent, intrusive thoughts or impulses that potentiate anxiety) and compulsions (*i.e.*, repetitive ritualistic physical or mental actions performed to reduce obsession-provoked anxiety [3,4,5]). While pharmacological and behavioral treatments relieve some patients of their OCD symptoms, up to 60% of patients go on to experience relapse [6]. Furthermore, 40%–60% of patients with OCD fail to fully respond to current treatments and remain resistant to treatment [7].

The most common first-line psychopharmacological treatments for OCD typically include preferential inhibitors of serotonin at the synapse, including clomipramine and selective-serotonin reuptake inhibitors (SSRIs) alone or in combination with cognitive behavioral therapy with exposure and response prevention [8]; for reviews see [9,10,11]. However, due to non-compliance and response-failure rates, novel treatment strategies including deep brain stimulation and repetitive transcranial stimulation (rTMS) are generating considerable interest [12,13]. As a preliminarily effective yet non-invasive neuromodulation technique, rTMS has shown some promise as a potential option for treatment-refractory patients [12]. To date, two randomized, sham-controlled studies using repeated low-frequency (1 Hz) stimulation of the supplementary motor area (SMA) in the treatment of refractory OCD [14,15] have been reported. While their sample sizes are relatively small, rTMS over SMA showed varying rates of clinical response, 42% [15] and 67% [14].

Currently, the etiology and pathophysiology of OCD are unknown. Core clinical features of OCD, however, have been associated with dysfunctional connectivity of the corticostriatal circuits [16,17]. Further evidence of network dysregulation in OCD is demonstrated by studies reporting dysregulation of neural oscillations during the resting state as well as during tests of cognitive function [18,19,20,21]. Key coordinators of neuronal activity across brain regions [22], neural oscillations are thought to be closely associated with cognitive function (for reviews see [23,24]). As rTMS has been shown to modulate delta and theta oscillation rhythms in healthy individuals [24], it may promote network level changes in OCD and reduce clinical symptoms.

Specifically, it has also been hypothesized that OCD and its associated clinical symptoms are the result of a deficit in inhibition throughout the cortico-striato-thalamo-cortical circuits [25,26,27,28]. Evidence suggests hyper-excitation within the network that includes the prefrontal and orbital frontal cortices, supplementary motor and premotor areas, the striatum, globus pallidus, and thalamus [29,30,31,32]. Mitigating cortical excitation using rTMS over the SMA has recently shown clinical utility in treating OCD: rTMS appears to restore some cortical inhibition and is correlated with effective clinical response [33,34].

Here, using a randomized double-blind study design, we investigated low-frequency (1 Hz) rTMS applied bilaterally and simultaneously over SMA for six weeks in patients with OCD from two treatment sites. We hypothesized that we would find significant improvement in patients’ clinical symptoms as classified by the Yale-Brown Obsessive Compulsive Scale (Y-BOCS). While this is not the first randomized double-blind clinical trial to assess the effects of rTMS over SMA to treat OCD symptoms [14,15], our results of a significant clinical response in the rTMS-treated group increase the generalizability of the treatment potential of rTMS in OCD. Furthermore, in light of the previous clinical trials, our findings also suggest that the number of rTMS treatments may contribute to optimal treatment efficacy, highlighting a possible “dosage” effect of rTMS.

## 2. Results

### 2.1. Repetitive Transcranial Stimulation (rTMS) Effects on Yale-Brown Obsessive Compulsive Scale (Y-BOCS)

Data were collected from 22 patients (Turkey, *n* = 7; Bulgaria, *n* = 15); in the overall sample, 11 (50%) were male and 11 (50%) were female. The average age in the group the received rTMS treatment (ACTIVE) was 33 ± 10 (mean ± SD) years and the group that received placebo rTMS (SHAM) 34 ± 14 (mean ± SD) years, and ranged from 18 to 54 years old. There were no significant differences in the distribution of sex or age between the Turkey and Bulgaria sites for the ACTIVE and SHAM groups (Table 1). Duration of OCD did, however, significantly differ between sites and treatment groups (Table 1): participants from Turkey had a significantly longer duration of OCD than those from Bulgaria (*F*(1,18) = 6.23, *p* = 0.022). This may represent a more treatment-refractory sample in Turkey that was thus less likely to respond to rTMS.

**Figure 1:**
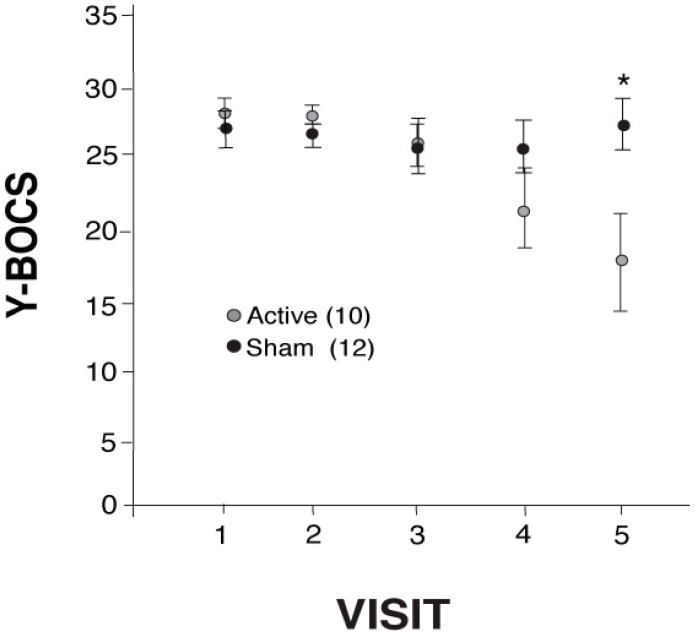
Yale-Brown Obsessive Compulsive Scale (Y-BOCS) mean scores (±SE) from baseline (Visit 1) to the end of repetitive transcranial magnetic stimulation (rTMS) treatment (Visit 5) with a significant difference (*) at Visit 5; * *p* < 0.05. Hedge’s *g* at Visit 5: 1.001.

**Figure 2:**
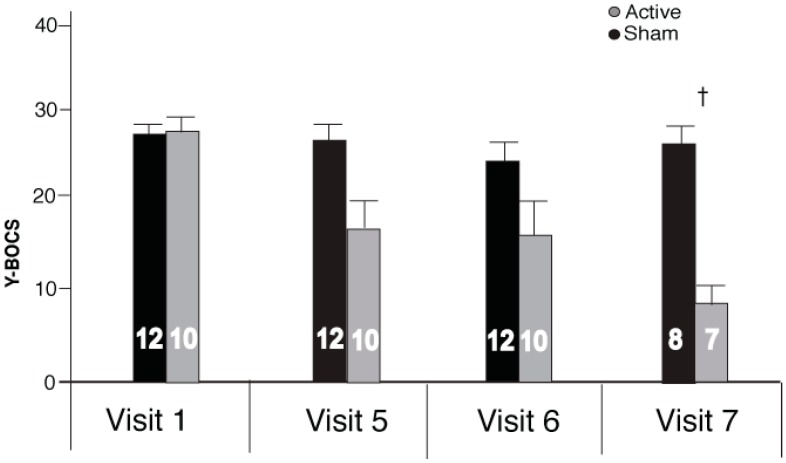
Y-BOCS mean scores (±SE) from baseline (Visit 1), the end of rTMS treatment (Visit 5), at the two-week (Visit 6) and six-week (Visit 7) follow-ups. Numbers inset in the bars represent participants in each group. † ACTIVE *vs.* SHAM *p* < 0.001.

Baseline Y-BOCS scores (Visit 1) did not significantly differ between Turkey and Bulgaria (no significant interaction for Site × Treatment (Table 1); nor a significant main effect of Site: *F*(1,18) = 0.091, *p* = 0.766). The active treatment group that received rTMS showed a clinically significant reduction in Y-BOCS scores across the five visits (before rTMS treatment (Visit 1) up to right after the last week of rTMS treatment (Visit 5), Figure 1; two-way ANOVA (Visit × treatment) revealed a significant difference between subjects as an effect of visit, *F*(1,20) = 11.86, *p* < 0.003, and within subjects Visit × treatment interaction, *F*(1,20) = 7.12, *p* < 0.015. There was no significant difference between treatment groups (*F*(1,20) = 0.84, *p* = 0.369). There was no significant decrease in the mean Y-BOCS scores for the SHAM group from Visit 1 to Visit 5 *t*(11) = −0.432, *p* = 0.674). Furthermore, on the first visit (baseline), mean Y-BOCS scores of ACTIVE *vs.* SHAM were not significantly different (*t*(20) = −0.626, *p* = 0.075). However, by Visit 5 (25 treatments) a significant effect of rTMS treatment became evident (*t*(20) = 2.46, *p* = 0.023). There was a similar reduction in obsessions (mean score reduction of 7 ± 5) and compulsions (mean score reduction of 8 ± 5) in the ACTIVE group following the 25 rTMS treatments (*t*(10) = −1.45, *p* = 0.187). Clinical response rate, defined as Y-BOCS reduction of ≥25% [35], was 80% (8 out of 10) in ACTIVE and 8% (1 out of 12) in SHAM. Of the 10 participants in the ACTIVE group, two from the Turkey site failed to respond to treatment (showed an increase in YBOCs scores), one of which dropped out after Visit 3 (two rTMS treatments). Of those who responded (*n* = 8), the ACTIVE treatment group showed an average 17 (±1.01) point reduction on the Y-BOCS (scoring from moderate-extreme scores at Visit 1 to subclinical-moderate by Visit 5). This represented a 40% mean reduction in YBOCS scores from baseline for the group receiving rTMS by the end of 25 rTMS treatments. Furthermore, at the two-week (Visit 6) follow-up visit (after the end of treatment), the Y-BOCS scores of the ACTIVE group continued to decline (Figure 2), but not significantly, demonstrating a sustained response to treatment (Visit 5 × Visit 6 × treatment: within group main effect of visit, *F*(1,20) = 4.32, *p* = 0.051; between group main effect of treatment, *F*(1,20) = 4.62, *p* = 0.044; but Visit 6 was not statistically different from Visit 5 for either group (ACTIVE (mean ± standard error (SE)): 16.9 ± 3.49, 15.6 ± 3.83 Visit 5 and 6, respectively, *t*(9) = 1.36, *p* = 0.207; SHAM (mean ± SE): 26.4 ± 2.03, 23.4 ± 2.49, Visit 5 and 6 respectively, *t*(11) = 1.76, *p* = 0.106)). At the six-week post-treatment follow-up (Visit 7), YBOCS scores for the participants at the Bulgarian site continued to decline slightly (Figure 2) but not significantly from Visit 6 (no significant difference between subjects as an effect of visit *F*(1,20) = 2.45, *p* = 0.141). However, at the six-week follow-up, YBOCS scores remained significantly reduced in the ACTIVE group (*t*(13) = 5.90, *p* < 0.001).

### 2.2. rTMS Effects on the Hamilton Depression Rating Scale 21 (HDRS-21) and Clinical Global Impression Scale (CGI)

Table 1 indicates that the HDRS-17 scores used to screen participants for major depression were not different between Turkey and Bulgaria. As shown in Table 2, both the HDRS-21 and CGI mean scores in the ACTIVE group decreased compared to SHAM by the end of rTMS treatment (Visit 5; HDRS-21: significant within subject interaction of visit × treatment *F*(1,20) = 7.90, *p* = 0.011; and a significant between-subject effect of treatment *F*(1,20) = 6.32, *p* = 0.021; CGI: a trend towards significance within subject effect of visit *F*(1,20) = 3.82, *p* = 0.065, a trend towards significance interaction of visit × treatment *F*(1,20) = 4.23, *p* = 0.053). Scores on both measures remained stable at the two-week follow-up (Table 2) but were no longer significantly different from SHAM.

## 3. Discussion

Following six weeks of rTMS, participants receiving the treatment showed a clinically significant decrease in reported OCD symptoms as measured by the Y-BOCS. Before rTMS these patients, despite concurrent pharmacological treatment, continued to score in the moderate to extreme range (23–35) of symptomatology on the Y-BOCS. Immediately after the full course of rTMS treatment, the scores in the ACTIVE group generally (with the exception of two participants that remained extreme) shifted to a less severe range, specifically sub-clinical to moderate (5–19). Furthermore, this effect was sustained: at a six-week follow-up visit, patients continued to report fewer OCD symptoms as the Y-BOCS scores remained statistically unchanged from those reported at the end of treatment. This study demonstrates that rTMS does have potential as an effective augmentative treatment in OCD.

The effect size found here (Hedge’s *g*: 1.00), while not quite as large, agrees with that of Gomes *et al.* [15] and Mantovani *et al.* [14], who found similar reductions in Y-BOCS scores in patients treated with low-frequency rTMS over the SMA (*n* = 12, *g* = 1.60; *n* = 9, *g* = 0.5, respectively). Like the findings reported here, Gomes *et al.* [15] reported at a three-month follow-up assessment (post-treatment) that scores remained unchanged. Our response rate was substantial; it was 80% compared to 67% [15] and 42% [14] in the two previous randomized sham-controlled trials [14,15]. It is possible that our response rate was influenced by placebo effects as rTMS administrators were not sufficiently blinded to treatment. However, across these three studies, response rates seem to correlate with the number of rTMS treatments and/or the rTMS treatment duration: two-weeks of 10 treatments yielded a 42% response rate [15]; four weeks of 20 treatments yielded a 67% rate [14]; and in the study reported here, six weeks of 25 treatments yielded an 80% response rate. All three studies (including this one) used 1 Hz, 20 min trains of rTMS bilaterally over the SMA. Our findings may suggest that a longer treatment window with more treatments could produce a better clinical effect.

A recent meta-analysis examining randomized and sham-controlled trials on rTMS for treating OCD concluded that low-frequency protocols targeting the orbitofrontal cortex or the supplementary motor area seem to be the most efficacious in treating OCD-related symptoms [12]. The presupplementary motor area is important for behavioral inhibition in healthy participants and the hyperactivity of this region has been correlated with deficits in response inhibition in patients with OCD [26]. The moderate success of the rTMS stimulation parameters has been postulated to be the result of the inhibitory effects of low-frequency rTMS on hyperactive orbitofronto-striatal and/or presupplementary motor circuits, the possible circuit that becomes dysfunctional to produce obsessions and compulsions of OCD [26]; for reviews see [28,34,36]. However, rTMS appears to be comparable in therapeutic efficacy to other augmentations strategies (e.g., antipsychotics [37]), suggesting that perhaps the optimum protocol for OCD has not yet been identified [12].

Active rTMS also significantly reduced symptoms of depression (HRDS-21). OCD and major depressive disorder (MDD) are frequently co-morbid psychiatric diagnoses [38]. Furthermore, low-frequency rTMS has also shown clinical efficacy in treating symptoms of treatment refractory MDD [39]. Here, active and sham rTMS treated participants all showed signs of mild depression, with all patient scores falling between 12 and 20 on the HRDS-21 with no incidence of MDD. Mantovani *et al.* [14] also found improvements in depression associated with rTMS to the SMA in the treatment of OCD. Because dysfunctional frontal-striatal circuits have been implicated in both disorders (for review see [36,40]), it is possible that improvement in depressive symptoms may have contributed to the improvement in OCD symptomology. However, using imaging studies, Remijnse *et al.* [41] have suggested that the cognitive inflexibility that characterizes OCD and MDD is associated with distinct neural correlates delineated by different frontal-striatal circuits, suggesting that improvement in depression may be secondary to rTMS’ effect on OCD [14]. The interplay of brain systems that underlies OCD and MDD co-morbidity requires further investigation.

There are a few important limitations to note in the study. Because of the SHAM method used here, it is possible that neither the rTMS administrator nor the patient may have been completely blind to the treatment conditions. Because SHAM individuals may have realized they were not receiving treatment, the potential placebo effects may have been mitigated; however, one of the SHAM-treated participants did show a significant treatment response, as reported on Y-BOCS. Furthermore, potential unblinding may have amplified the differences in Y-BOCS between the SHAM and ACTIVE groups. It is also unfortunate that the six-week follow-up data was only collected for the Bulgarian site. This limits the extent to which we can generalize the duration of rTMS’ effects. Finally, it is possible that patients from Turkey and Bulgaria represented two different populations, as the Turkey sample showed significantly more years of OCD than the Bulgarian sample. The relatively small sample size is likely responsible for this difference. Whether or not duration of an OCD illness limits or potentiates therapeutic efficacy of rTMS is not known.

## 4. Methods

### 4.1. Patients

Participants aged 18 to 65 were recruited from three different mood disorder sites: Kingston, Ontario; Izmir, Turkey; and Sofia, Bulgaria. Subsequently one site dropped out of the study due to technical difficulties (Kingston). Thus, the data reported here are only from the Turkish and Bulgarian sites. Written informed consent was obtained from patients before beginning any study-related procedure, in accordance with two ethics committees: Turkey, Dokuz Eylül University Ethics Committee; Bulgaria, Ethics Committee for Multicenter Trials of Ministry of Health. Treatment randomization was performed by an individual who received enrollment logs and enrollment envelopes that were randomly assigned to patient enrollment and had protocol allocation indicated by a letter designation (C: ACTIVE; D: SHAM). The clinical raters and rTMS administrators were blinded to the randomization procedure. Clinical raters and rTMS administrators were separate individuals. Clinical diagnoses were determined by a blinded psychiatrist using the Diagnostic and Statistical Manual of Mental Disorders, Fourth Edition (MINI) criteria for primary Obsessive Compulsive Disorder (OCD). rTMS administrators were instructed to provide the protocol indicated by the letter allocated to the patient: the intensity for both C and D protocols (active and sham, respectively) remained the same; however, positioning of the coil over the target zone differed.

Participants who had primary OCD and eight weeks of adequate treatment and four weeks of a stable dose of SSRI but were not responding (treatment refractory) with a score of at least 20 on the Y-BOCS were included in this study. Current medication regimes were followed throughout the treatment. Benzodiazepines (Lorazepam or Diazepam) were also sustained in case of severe anxiety, as these medications do not change the motor threshold.

Study exclusion criteria included: a diagnosis of schizophrenia, current major depressive disorder (Hamilton Depression Rating Scale 17 item (HDRS-17) > 18), other psychotic disorders, bipolar I disorder, or substance and alcohol dependence within the last six months; severe axis II disorder; suicidal (score ≥ 6, moderate or severe stage in MINI); metallic implant in the cranium (except mouth); severe or unstable medical conditions; failure to respond to electroconvulsive therapy or have had transcranial magnetic stimulation treatment in the past six months; history of epilepsy; neurological disorders leading to increased intracranial pressure; and severe cardiac disorder and/or with intracardiac lines or cardiac pacemakers.

In total, 23 participants were enrolled in the study. Of these, 20 participants completed all 25 rTMS treatments. One participant was not included in the data analysis as the participant withdrew after the first visit. In total, 22 participants were randomly assigned to the SHAM group (*n* = 12) and ACTIVE (*n* = 10) groups. One patient completed only 20 rTMS sessions and then withdrew; this patient was included in the final analysis. A second patient did not return for the two-week follow-up visit (Visit 6) but was also included in the analysis. Almost all patients were on at least one medication during the duration of the study: one patient was drug free and six were receiving only one medication (both in the active group); the remainder of patients in both sham and active groups received polypharmacotherapy (two or more concurrent medication). Patients received SSRIs (Fluvoxamine (1 ACTIVE), Fluoxetine (SHAM: 1), Paroxetine (ACTIVE: 2; SHAM: 1), Sertraline (ACTIVE: 2; SHAM: 7), Citaloropram (ACTIVE: 3), Escitalopram (ACTIVE: 1; SHAM: 3), non-benzodiazepine hypnotics (Zilpidem (ACTIVE: 3), Zopiclone (ACTIVE: 1)), tricyclic antidepressants (Clomipramine (ACTIVE: 2)), typical antipsychotics (Flupentixol (ACTIVE: 2; SHAM: 3), Flaunxol (ACTIVE: 1)), tetracyclic antidepressants (Mirtazapine (ACTIVE: 1)), neuroleptics (Promethazine (ACTIVE: 1)), atypical antipsychotics (Quetiapine (ACTIVE: 1), Ziprazidone (SHAM: 1), Risperidone (SHAM: 1)), antiparkinsononian anticholinergics (Biperiden (SHAM: 1)), anticonvulsants (Etifoxine (SHAM: 1)), and melatonin (SHAM: 1)).

### 4.2. Stimulation Parameters

Active group participants received low-frequency active rTMS (1 Hz, 110% of the Resting Motor Threshold (RMT), for 20 min (5 min trains with 2 min intertrain intervals) by figure eight shaped coils; Medtronic MagPro R30, Farum, Denmark) bilaterally to Supplementary Motor Area (SMA) five sessions a week for the first four weeks. During the fifth week, sessions were reduced to three times per week and again to twice a week during the sixth week. Sham group patients received a coil that was held 90 degrees from the skull, with an intensity of 110% of RMT. The remaining procedure was the same as the active group. Participants in the Istanbul, Turkey site did not participate in the six-week follow-up after the end of treatment.

The stimulus site was 15% of the distance between inion and nasion, anterior to vertex (Cz), which corresponds to the bilateral SMA according to the 10–20 International EEG localization system. The RMT was determined using single-pulse TMS, given stimuli localizing the coil to the left side of the skull matching to the right hand area according to 10–20 EEG localization system.

### 4.3. Outcome Measures

The standardized clinical rating scales—Yale–Brown Obsessive Compulsive Scale (Y-BOCS), Clinical Global Impression Scale (CGI), and Hamilton Depression Rating Scale (HDRS-21)—were administered by a blinded clinical rater and used to measure the treatment outcomes.

A total of seven visits for the Bulgarian group and six visits for the Turkey group were required per participant to measure treatment outcomes: The scales were administered and evaluated by a blind rater at the pretreatment baseline phase (Visit 1), biweekly during the treatment phase (Day 1 and two, four, and six weeks after the start of treatment date) and at two and six weeks (Bulgarian group only) after the end of treatment.

### 4.4. Statistical Analysis

Analyses were performed using SPSS version 23 (Chicago, IL, USA). Comparisons between sites (Turkey × Bulgaria) for treatment (ACTIVE × SHAM) were made using two-way (Site × Treatment) analysis of variance (ANOVA). One-way ANOVA examined significant interactions. Pooling data across site, the effect of TMS treatment on outcome measures was analyzed by comparing treatment (ACTIVE *vs.* SHAM) across visits (seven sessions) using a repeated measures ANOVA. A two-way ANOVA (Treatment *vs.* Visit) examined the main effects and interactions. Missing data (for two participants in the ACTIVE group from the Turkey site) were replaced with the last observation carried forward in order to perform intention-to-treat methodology. Independent samples *t*-test and paired *t*-test were used to explore significant interactions. When repeated measures were performed, trend analysis (contrasts) was reported in every case. Neither the sphericity of variance nor a significant main effect of the within-subject variable is an assumption of running trend analysis. Hedge’s *g* was used to report the effect size (magnitude of the difference) following the 25 treatments (Visit 5).

## 5. Conclusions

Here we demonstrate that low-frequency rTMS applied bilaterally to the SMA significantly improves clinical symptoms of OCD, an effect that was sustained for at least six weeks following the end of treatment. Given the low side-effects profile, future studies should examine drug-naïve patients and other stimulation parameters [42]. Furthermore, future studies could also extend the follow-up period beyond three months to determine the duration of clinical efficacy.

## Figures and Tables

**Table 1:** *p*-Values for the interaction of site (Bulgaria *vs.* Turkey) by treatment (ACTIVE *vs.* SHAM). Duration of obsessive compulsive disorder (OCD) in years (y) and baseline Hamilton Depression Rating Scale 17 (HDRS-17) and Yale-Brown Obsessive Complusive Scale (Y-BOCS) scores for each site are listed. *p*-Values are from two-way analysis of variance (ANOVA). Bolded *p*-values were followed up with *post hoc* analysis.

Samples	Bulgaria	Turkey	ANOVA
Male (age ± SD)	7 (28 ± 12)	4 (28 ± 7)	*p* = 0.154 (for age, sex collapsed)
Female (age ± SD)	8 (38 ± 11)	3 (43 ± 16)	–
Duration of OCD (y ± SD)	7 ± 7	18 ± 12	***p* = 0.037**
Baseline (Visit 1) HDRS-17	13 ± 1	12 ± 4	*p* = 0.104
Baseline (Visit 1) Y-BOCS	28 ± 5	28 ± 4	*p* = 0.847

**Table 2:** Independent samples *t*-test results for the Hamilton Depression Rating Scale 2 (HDRS-21) and the Clinical Global Impression Scale (CGI) mean (±SD) scores for ACTIVE *vs.* SHAM.

Samples	Active (Mean ± SD)	Sham (Mean ± SD)	*t*-Test
HDRS-21			
Baseline (Visit 1)	14 ± 3	15 ± 2	*p* = 0.437
Treatment end (Visit 5)	8 ± 8	17 ± 6	*p* = 0.012
Two-week follow-up (Visit 6)	9 ± 10	16 ± 6	*p* = 0.084
CGI			
Baseline (Visit 1)	5 ± 1	5 ± 1	*p* = 1.000
Treatment end (Visit 5)	4 ± 2	5 ± 2	*p* = 0.084
Two-week follow-up (Visit 6)	4 ± 2	5 ± 2	*p* = 0.084
